# Preparation and Electrochemical Properties of Li_3_V_2_(PO_4_)_3−*x*_Br*_x_*/Carbon Composites as Cathode Materials for Lithium-Ion Batteries

**DOI:** 10.3390/nano7030052

**Published:** 2017-02-24

**Authors:** Xiaoyu Cao, Lulu Mo, Limin Zhu, Lingling Xie

**Affiliations:** 1 School of Chemistry and Chemical Engineering, Henan University of Technology, Zhengzhou 450001, China; molulu_1989@126.com (L.M.); linglingxie51@163.com (L.X.); 2 Key Laboratory of High Specific Energy Materials for Electrochemical Power Sources of Zhengzhou City, Henan University of Technology, Zhengzhou 450001, China

**Keywords:** lithium-ion batteries, Li_3_V_2_(PO_4_)_3_/carbon composites, cathode materials, bromine ion doping, enhanced electrochemical performances

## Abstract

Li_3_V_2_(PO_4_)_3−*x*_Br*_x_*/carbon (*x* = 0.08, 0.14, 0.20, and 0.26) composites as cathode materials for lithium-ion batteries were prepared through partially substituting PO_4_^3−^ with Br^−^, via a rheological phase reaction method. The crystal structure and morphology of the as-prepared composites were characterized by X-ray diffraction (XRD) and scanning electron microscopy (SEM), and electrochemical properties were evaluated by charge/discharge cycling and electrochemical impedance spectroscopy (EIS). XRD results reveal that the Li_3_V_2_(PO_4_)_3−*x*_Br*_x_*/carbon composites with solid solution phase are well crystallized and have the same monoclinic structure as the pristine Li_3_V_2_(PO_4_)_3_/carbon composite. It is indicated by SEM images that the Li_3_V_2_(PO_4_)_3−*x*_Br*_x_*/carbon composites possess large and irregular particles, with an increasing Br^−^ content. Among the Li_3_V_2_(PO_4_)_3−*x*_Br*_x_*/carbon composites, the Li_3_V_2_(PO_4_)_2.86_Br_0.14_/carbon composite shows the highest initial discharge capacity of 178.33 mAh·g^−1^ at the current rate of 30 mA·g^−1^ in the voltage range of 4.8–3.0 V, and the discharge capacity of 139.66 mAh·g^−1^ remains after 100 charge/discharge cycles. Even if operated at the current rate of 90 mA·g^−1^, Li_3_V_2_(PO_4_)_2.86_Br_0.14_/carbon composite still releases the initial discharge capacity of 156.57 mAh·g^−1^, and the discharge capacity of 123.3 mAh·g^−1^ can be maintained after the same number of cycles, which is beyond the discharge capacity and cycleability of the pristine Li_3_V_2_(PO_4_)_3_/carbon composite. EIS results imply that the Li_3_V_2_(PO_4_)_2.86_Br_0.14_/carbon composite demonstrates a decreased charge transfer resistance and preserves a good interfacial compatibility between solid electrode and electrolyte solution, compared with the pristine Li_3_V_2_(PO_4_)_3_/carbon composite upon cycling.

## 1. Introduction

Lithium-ion batteries (LIBs) as advanced electrochemical power sources are considered to be the ideal choice for numerous portable consumer electronics, such as smartphones, tablets, notebook PCs, and camcorders, due to their high energy density (both volumetric and gravimetric), low self-discharge rate, wide operating temperature range, lack of a memory effect, and environmental friendliness. Especially in recent years, LIBs have been regarded as the most promising power sources for hybrid electric vehicles (HEVs) and electric vehicles (EVs), which require very high energy and power densities for LIBs [1,2,3,4,5,6,7]. However, LIBs still have some problems, such as an unsatisfactory energy density, high cost, and safety risk. In particular, the energy density of LIBs cannot support the longer driving range of EVs. At present, to a great extent, the electrochemical properties of LIBs are dominated by the electrochemical performances of cathode materials. Therefore, it is of great importance to develop new cathode materials with a large capacity, high working voltage, excellent safety, good cycling life, and low-cost [8]. Among the vast number of reported cathode materials, NASICON-structured monoclinic Li_3_V_2_(PO_4_)_3_ (LVP) has drawn much attention, because of an average 4.0 V (~0.6 V higher than LiFePO_4_) extraction/reinsertion voltage obtained between 3.0 and 4.8 V, a higher theoretical capacity of 197 mAh·g^−1^ for the complete removal of three Li^+^ ions [9,10], and a low cost. However, the main drawback of pure LVP is its very low intrinsic electronic conductivity, which causes high electrode polarization and restricts its application in the field of dynamic batteries [11,12,13].

To overcome this problem, strenuous efforts have been devoted to improving the electronic conductivity of LVP, including lattice doping with metal ions [10,14,15,16,17], surface coating with carbon sources [18,19,20] or high electrical conductivity metal oxides [21,22,23], reducing particle size [24,25], and controlling particle morphologies [26,27,28]. Among these modified techniques, it is common to combine carbon with high electron conductivity and form Li_3_V_2_(PO_4_)_3_/carbon (LVPC) composites. On this basis, it is preferable to dope LVP with trace elements to produce lattice defects and further enhance its intrinsic conductivity. Over the years, studies relating to cation doping have been extensively explored, such as V-site substitutions with Zn^2+^ [10], Mg^2+^ [29], Fe^2+^/Fe^3+^ [15,30], Cr^3+^ [31], Co^2+^ [32], Ce^3+^ [16], Al^3+^ [17], and La^3+^ [33], and Li-site substitutions with Na^+^ [34,35] and K^+^ [14]. However, anion doping has been less often attempted and it is mainly substituted at the polyanion PO_4_^3−^-site of LVP. Zhong et al. [36] have synthesized Li_3_V_2_(PO_4_)_3−*x*_F*_x_*/carbon (*x* = 0, 0.05, 0.10, and 0.15) composites, in which PO_4_^3−^ was partially substituted with F^−^, through a sol-gel synthesis method. The Li_3_V_2_(PO_4_)_3−*x*_F*_x_*/carbon composite showed a good electrochemical performance with an initial discharge capacity of 117 mAh·g^−1^ at the current rate of 10 *C* in the voltage range of 3.0–4.2 V and a capacity retention of 90.6% after 30 cycles. Yan et al. [37] adopted Cl^−^ doping and prepared Li_3_V_2_(PO_4_)_3−*x*_Cl*_x_*/carbon composites, in which PO_4_^3−^ was partially substituted with Cl^−^. In their work, the Li_3_V_2_(PO_4_)_2.88_Cl_0.12_/carbon composite yielded a discharge capacity as high as 106.9 mAh·g^−1^ after 80 cycles at the current rate of 8 *C* in the voltage range of 3.0–4.3 V. The reports mentioned above show that anion doping is an effective way to improve the electrochemical properties of LVP. However, to the best of our knowledge, there are no reports concerning Br^−^-doped LVP. In fact, the radius of Br^−^ is 196 pm, which is much closer to that of PO_4_^3−^ (236 pm), compared to the radius of F^−^ and Cl^−^. Hence, it may be more suitable to partially replace the PO_4_^3−^ with Br^−^ in LVP, and prepare the Li_3_V_2_(PO_4_)_3−*x*_Br*_x_*/carbon (LVPBC) composites with enhanced electrochemical properties.

In the present work, a series of Br^−^-doped LVPC composites were synthesized through the rheological phase reaction method. A comparison of the LVPBC composites with the pristine LVPC composite, and the effects of Br^−^-doping on the crystal structure, morphology, and electrochemical properties of LVPC, have been studied in detail.

## 2. Experimental

The LVPBC composite cathode materials were prepared by the rheological phase reaction method, in a similar manner to those reported in previous references [38,39,40]. All of the chemicals used in this work were of an analytical grade, without any pre-treatment. Firstly, stoichiometric amounts of LiOH·H_2_O, V_2_O_5_, NH_4_H_2_PO_4_, LiBr, and C_6_H_8_O_7_·H_2_O (3:1:(3−*x*):*x*:2) as raw materials, were mixed thoroughly by grinding in an agate mortar. Secondly, the solid-liquid rheological body (muddy state) was obtained by adding a proper amount of deionized water. Next, the rheological body was transferred to a cylindrical Teflon-lined stainless autoclave. Thirdly, the sealed autoclave was humidified at 80 °C in a blast oven for 6 h and then dried at 100 °C for another 10 h, to obtain pale blue precursor. Afterwards, the obtained precursor was carefully ground and pre-heated at 350 °C for 3h under a flowing argon atmosphere in a tube furnace, followed by a slow cool to room temperature. Finally, the preheated powder was reground and sintered at 800 °C for 8 h under the same atmosphere, to yield a series of LVPBC (Li_3_V_2_(PO_4_)_3−*x*_Br*_x_*/carbon, *x* = 0.08, 0.14, 0.20, and 0.26) composites. For convenience, the four Br^−^-doped LVPBC composites are referred to as LVPBC-0.08, LVPBC-0.14, LVPBC-0.20, and LVPBC-0.26, respectively. In order to investigate the effect of Br^−^ doping, the pristine LVPC composite was also synthesized under the same conditions, but without the addition of LiBr powder. According to our previous study [39,41], the carbon content in a LVPC composite is around 15 wt %.

The structure of the as-prepared samples were characterized by X-ray diffraction (XRD), using a Rigaku D/Max-2500 X-ray diffractometer (Rigaku Corporation, Tokyo, Japan) with a Cu-Kα radiation source in the 2θ range of 10°−80°. The surface morphologies of the as-prepared composites were observed using a JSM-6510LV scanning electron microscope (SEM, JEOL Ltd, Tokyo, Japan).

The galvanostatic charge/discharge tests were evaluated using2016 coin-type cells. The cathode electrodes were fabricated by roll-pressing the as-synthesized active powder, super carbon, and PTFE microemulsion (60 wt %) ata weight ratio of 80:10:10, into a thick film, and then pressing the film onto an aluminum current collector (pressure of 20 MPa). Lithium metal was used as a counter-electrode, the commercial polyethylene (PE) film (ND420 H129-100, Asahi Kasei Chemical Co., Osaka, Japan) as a separator, and the 1M LiPF_6_ in Ethylene Carbonate (EC) and Dimethyl Carbonate (DMC) (1:1, by volume ratio, provided by Zhangjiagang Guotai-Huarong New Chemical Materials Co., Ltd., Suzhou, China) as an electrolyte. The cells were assembled in an argon-filled glove box (JMS-3, Nanjing Jiumen Automation technology Co., Ltd., Nanjing, China). The charge/discharge cycles were performed utilizing a multi-channel CT-3008W-5V5mA-S4 battery tester (Shenzhen Neware Electronics Co., Ltd., Shenzhen, China) between 3.0 and 4.8 V at room temperature, with different current densities. Electrochemical impedance spectroscopy (EIS) was recorded using a CHI660D electrochemical workstation (Shanghai Chenhua Co., Ltd., Shanghai, China) at a frequency range of 100 kHz to 10 mHz, with a potential amplitude of ±5 mV. In order to detect changes in the crystal structure of the LVPBC-0.14 composite electrode during cycling, ex-situ XRD examination was performed at the states of fully discharged to 3.0 V vs. Li^+^/Li. Fully discharged coin cells were allowed to equilibrate before they were moved to an argon filled glove box, where the electrodes were removed from the cells and rinsed with DMC solvent, in order to remove any residual salt.

## 3. Results and Discussion

**Figure 1:**
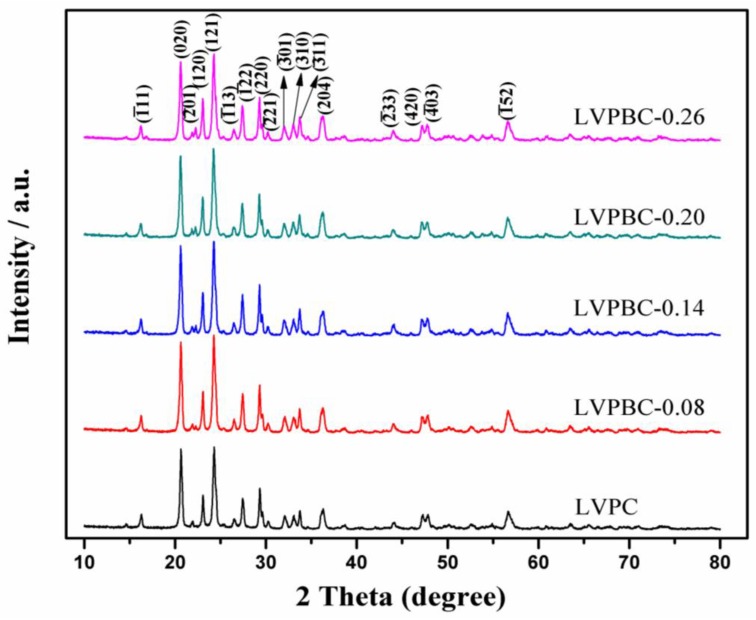
X-ray diffraction (XRD) patterns of the LVPBC and pristine LVPC composites.

Figure 1 shows the XRD patterns of the pristine LVPC composite and LVPBC composites with various Br^−^-doping amounts. As illustrated in Figure 1, the diffraction peak positions of the LVPBC composites are almost identical to the pristine LVPC. However, the Bragg diffraction peaks of the LVPBC composites become sharper, indicting the good crystallinity for the as-synthesized LVPBC composites. No impurity peaks are detected in the XRD patterns, which indicates that a small amount of Br^−^-doping does not change the basic crystal structure [39]. But then, the main diffraction peak intensities of the LVPBC composites are heightened. The reason for this may be that Br^−^ ions enter into the lattice of LVP and form a solid solution, which brings about a certain effect on the crystal microstructure. In addition, no peaks of carbon can be seen in XRD patterns, which may be ascribed to its amorphous structure. The lattice parameters of the pristine LVPC and LVPBC composite are calculated by Jade software refinement and listed in Table 1. From Table 1, it can be seen that the cell volume of LVPBC-0.08 and LVPBC-0.14 composites shrunk, when compared with the pristine LVPC composite. This may be consistent with the fact that the radius of Br^−^ (196 pm) is smaller than that of PO_4_^3−^ (238 pm) [15]. However, the cell volume of the LVPBC-0.20 composite is larger than that of the undoped one, which has the appearance of mixed valence of V^3+^/V^2+^ ions after partial substitution of PO_4_^3−^ ions with Br^−^ ions, due to the requirement of electrical neutrality [32,42]. Because the radius of V^2+^ is larger than that of V^3+^, the cell volume of the LVPBC-0.20 composite is expanded. Due to the common effect by these two opposite factors, the cell volume of theLVPBC-0.26 composite begins to contract, in comparison to the pristine LVPC composite.

**Figure 2:**
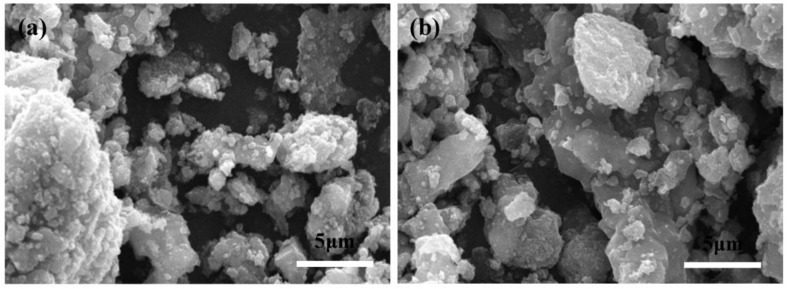

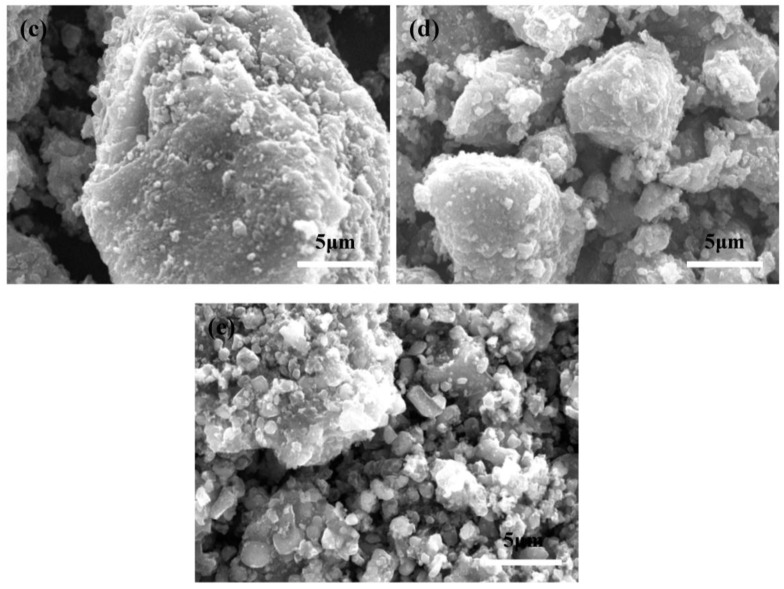
Scanning electron microscopy (SEM) images of the LVPBC and pristine LVPC composites. LVPBC-0.08 (**a**); LVPBC-0.14 (**b**); LVPBC-0.20 (**c**); LVPBC-0.26 (**d**); and LVPC (**e**).

Figure 2 shows the SEM images of the LVPBC and pristine LVPC composites. As shown in Figure 2, the pristine LVPC composite is composed of regular particles with small particle size. However, the particles of the LVPBC composites become inhomogeneous and irregular, and the particle size gradually increases with an increase in the Br^−^-doping concentration. In terms of the XRD patterns, the higher the Br^−^-doping amount is, the stronger the intensity of the diffraction peak is, and the bigger the particle size is. Therefore, the SEM results reveal that Br^−^-doping can increase the particle size of LVPBC composites, and are in accordance with the XRD patterns in Figure 1 and the previous literature [36].

**Figure 3:**
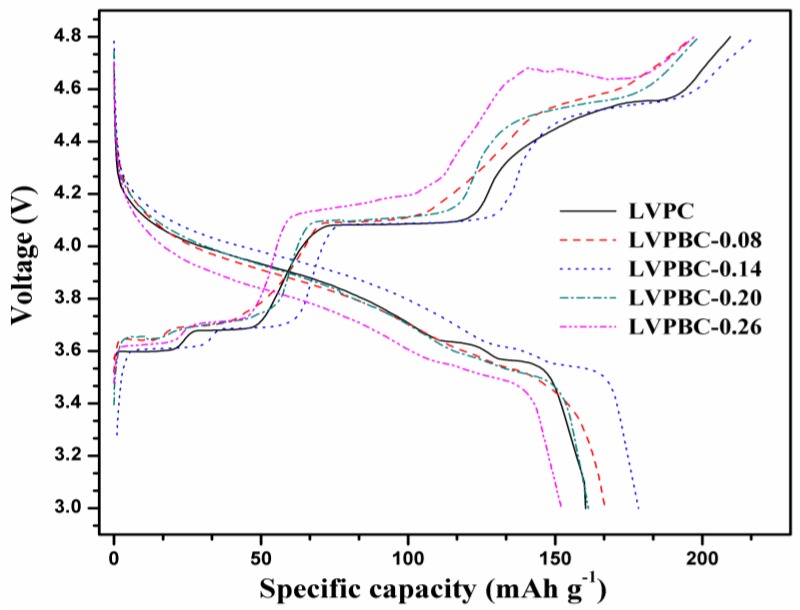
Initial charge/discharge curves of the LVPBC and pristine LVPC composites.

Figure 3 presents the initial charge/discharge curves of the LVPBC and pristine LVPC composites at the current rate of 30 mA·g^−1^ between 3.0 and 4.8 V. As illustrated in Figure 3, the initial discharge capacities of the LVPBC-0.08, LVPBC-0.14, LVPBC-0.20, LVPBC-0.26, and pristine LVPC composites, are 166.95, 178.33, 161.31, 152.11, and 160.39 mAh·g^−1^, respectively. Obviously, the LVPBC-0.14 composite exhibits the highest discharge capacity. Although the substitution of Br^−^ with PO_4_^3−^ causes the cell volume of the LVPBC-0.14 composite to decrease, the lattice volume of the LVPBC-0.14 composite still reaches 871.84 Å^3^, where the Li^+^ ions can diffuse relatively freely. On the other hand, it is generally believed that the occurrence of a mixed valence of V^3+^/V^2+^ in the LVPBC-0.14 composite can enhance its electronic conductivity [32]. All of what is mentioned above results in the lowest charge plateau, highest discharge plateau, and maximal discharge capacity for the LVPBC-0.14 composite, compared to all of the other composites. However, it is found that the discharge capacities of the LVPBC composites are not monotonously raised with an increasing Br^−^-doping content. A decreased discharge capacity is observed for the LVPBC-0.26 composite, compared with the pristine LVPC composite. The possible reason for this is that excessive Br^−^-doping leads to a large particle size in the LVPBC-0.26 composite (Figure 2), which extends the transport length for Li^+^ ions and electrons during the charge/discharge process and elevates the electrode polarization. In the mean time, the shrinkage of the cell volume is not conducive to moving the Li^+^ ions in the solid phase, thus reasonably resulting in the highest charge platform, lowest discharge platform, and minimal discharge capacity for the LVPBC-0.26 composite. As a result, the appropriate Br^−^-doping amount is essential for improving the electrochemical performance of the LVPC composite.

**Figure 4:**
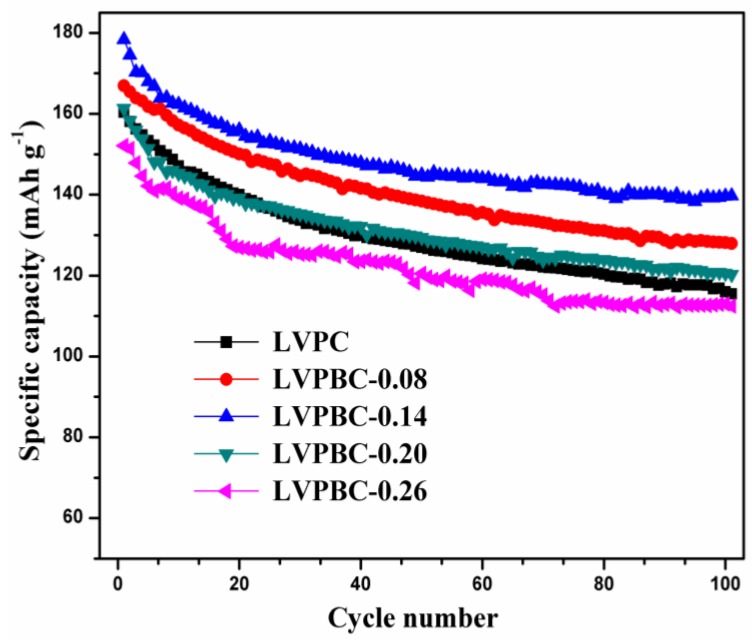
Cycling performance of the LVPBC and pristine LVPC composites at the current density of 30 mA·g^−1^ in the voltage range of 3.0–4.8 V.

Figure 4 shows the cycling performance of the LVPBC and pristine LVPC composites, at the current rate of 30 mA·g^−1^ in the voltage range of 3.0–4.8 V within 100 cycles. It is observed that the LVPBC-0.14composite demonstrates the highest discharge capacity and the best cycle stability. Its initial discharge capacity reaches 178.33 mAh·g^−1^ and the discharge capacity of 139.66 mAh·g^−1^ is still retained after 100 cycles. Moreover, apropos of the LVPBC-0.08 composite, it manifests an excellent cycle performance, which is second only totheLVPBC-0.14 composite. Although the discharge capacity of the LVPBC-0.20 composite is almost equal to that of the pristine LVPC composite in the first few cycles, its discharge capacity surpasses that of the latter after 30 cycles. However, as for the LVPBC-0.26 composite, its discharge capacity and cycling performance are inferior to those of the pristine LVPC composite, which should be attributed to its larger particle size and smaller cell volume, and which is unfavorable to the movement of Li^+^ ions. In addition, when the cell is charged to the high voltage of 4.8 V, the appearance of the high oxidizing V^5+^ ions makes LVPC more vulnerable to side reactions with the electrolyte [13,43]. After Br^−^-doping, the number of V^5+^ ions will decrease upon charging, due to the requirement of electronical neutrality, which decreases the probabilities of a side reaction between the LVPBC composites and the electrolyte to a certain extent, thus improving the cycling performance of the LVPBC composites, with the exception of the LVPBC-0.26 composite.

**Figure 5:**
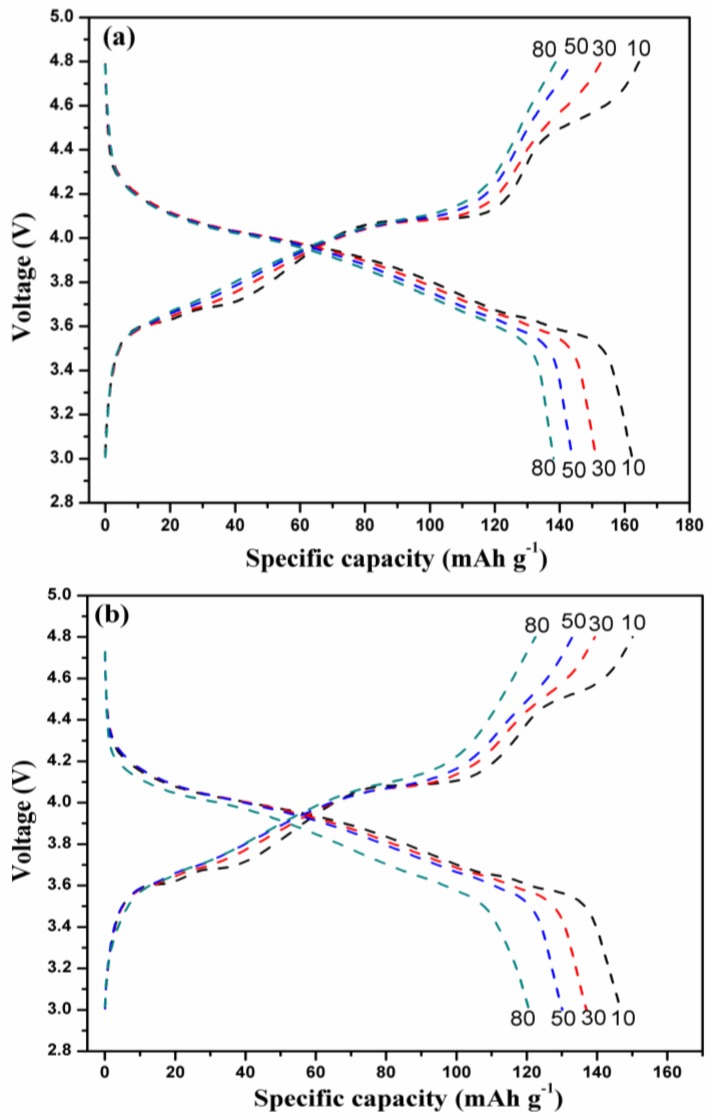
Selected charge/discharge curves of the LVPBC (**a**) and pristine LVPC (**b**) composites at the current density of 30 mA·g^−1^ in the voltage range of 3.0–4.8 V.

Comparisons of the charge/discharge curves of the LVPBC-0.14 composite, together with those of the pristine LVPC composite at the 10th, 30th, 50th, and 80th cycle at the current density of 30 mA·g^−1^ in the voltage range of 3.0–4.8 V, are shown in Figure 5. As depicted inthe selected charge/discharge curves, the shape of the charge/discharge curves of the LVPBC-0.14 composite is basically consistent with those of the pristine LVPC composite, and there are only differences in the length of charge/discharge voltage plateaus at the different cycles, demonstrating that Br^−^-doping does not change the phase transition of the LVPBC composite during the charge/discharge process [37]. With ongoing cycling, the discharge capacity of both of the composites gradually decays. Accordingly, the charge/discharge voltage platforms of the two composites are gradually shortened. However, the discharge capacity of the LVPBC-0.14 composite still attains 162.36, 151.27, 143.88, and 138.07 mAh·g^−1^ at the 10th, 30th, 50th, and 80th cycle, respectively. By contrast, the pristine LVPC composite only delivers a discharge capacity of 146.78, 136.96, 130.08, and 120.65 mAh·g^−1^, respectively, at the same cycle. It is clearly seen in Figure 5 that the LVPBC-0.14 composite exhibits a high discharge capacity and excellent cycle performance after Br^−^-doping, which is ascribed to a reduction in the resistance for Li^+^ ions intercalation/de-intercalation in the LVPBC-0.14 composite, and will be evidenced in the following EIS results.

**Figure 6:**
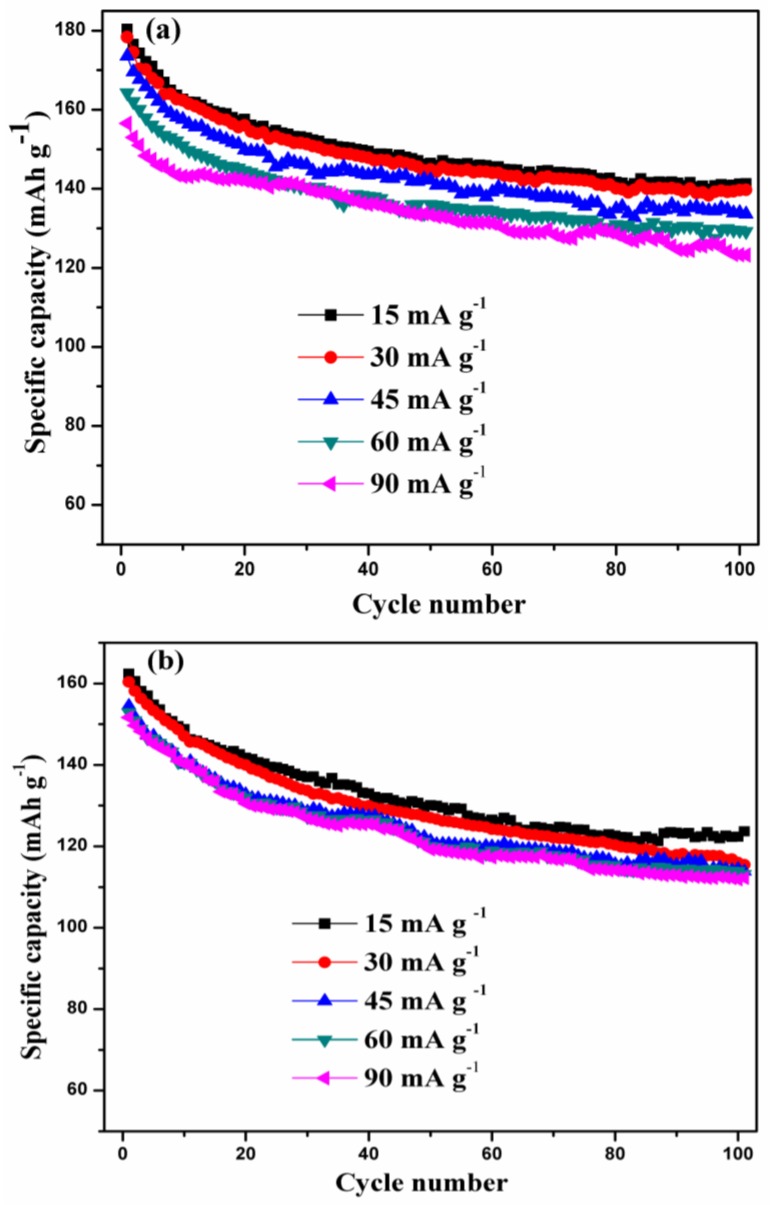
Rate capabilities of the LVPBC (**a**) and pristine LVPC (**b**) composites in the voltage range of 3.0–4.8 V.

Figure 6 presents the rate capabilities of the LVPBC-0.14 and pristine LVPC composites in the voltage range of 3.0–4.8 V, at the different current densities. It can be seen that the LVPBC-0.14 composite reveals a great improvement in both the discharge capacity and cycle performance, compared with the pristine LVPC composite. For instance, the first discharge capacities of the LVPBC-0.14 composite amount to 180.31, 178.33, 173.61, 164.28, and 156.57 mAh·g^−1^ at the current densities of 15, 30, 45, 60, and 90 mA·g^−1^, respectively. Conversely, those of the pristine LVPC composite are 162.41, 160.39, 154.54, 152.90, and 151.67 mAh·g^−1^, under the same conditions. After 100 cycles, the discharge capacities of the LVPBC-0.14 composite still deliver 141.23, 139.66, 133.97, 129.48, and 123.27 mAh·g^−1^, respectively, whereas the pristine LVPC composite only yields 122.36, 116.00, 114.41, 113.93, and 111.87 mAh·g^−1^. Overall, the LVPBC-0.14 composite manifests a desirable rate capability, indicating that Br^−^-doped LVPC composites are suitable as high-rate capability cathode materials in LIBs.

**Figure 7:**
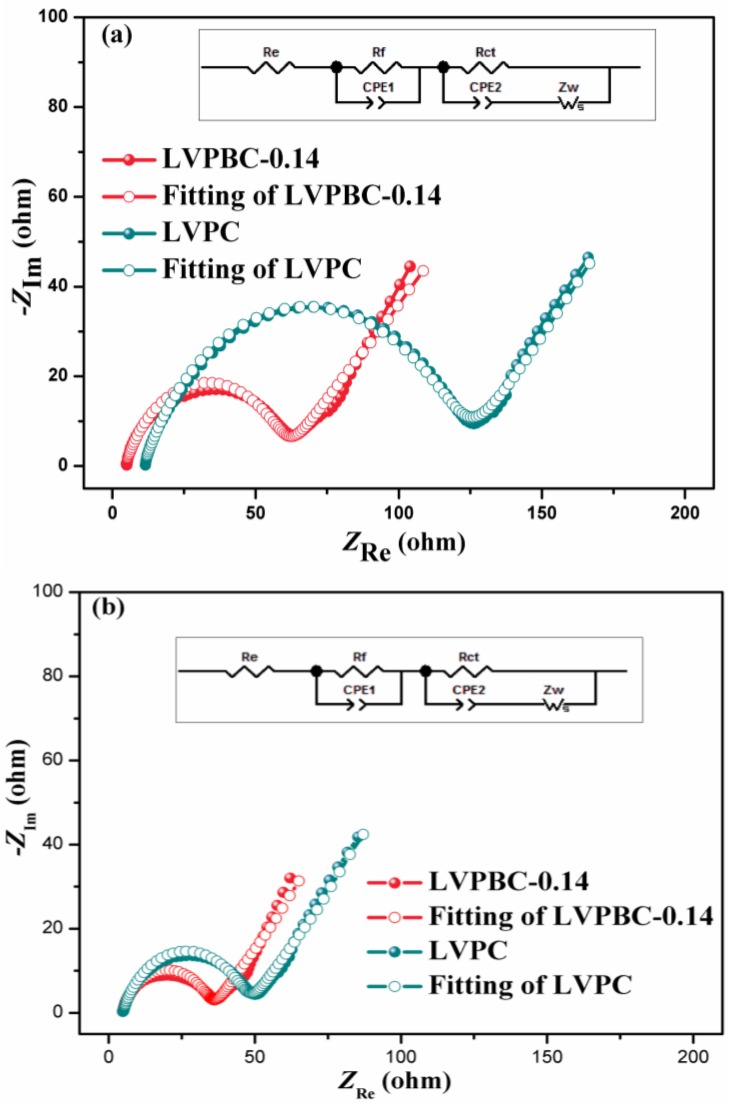
Electrochemical impedance spectroscopy (EIS) of the LVPBC and pristine LVPC composites under fully discharged state at the 10th (**a**) and 20th (**b**) cycles.

EIS were measured to understand the kinetic process of the LVPBC-0.14 and pristine LVPC composites under a fully discharged state at the 10th and 20th cycles. The Nyquist plots of the two composites are shown in Figure 7. All of the spectra are composed of a semicircle in the high and middle frequency regions, and an inclined line in the low frequency region. It is well known that the semicircleis related to the charge transfer resistance (*R*_ct_) between the electrode and electrolyte interface, while the sloping line in the low frequency is the Warburg impedance (*Z*_w_), attributed to the Li^+^ ion diffusion in the solid electrode [44,45]. The equivalent circuit is inserted into Figure 7, where *R*_e_ and *R*_f_ stand for the resistance of the whole reaction system, including the electrolyte resistance, interparticle contact resistance, and other physical resistances between the electrode and electrolyte interface, and the CPE (constant phase element) is a double layer capacitance on the electrode surface. By fitting EIS of these two composites at the same cycle, it is found that *R*_ct_ of the LVPBC-0.14 composite is much smaller than that of the pristine LVPC composite, indicating that the charge transfer reaction is more favorable on the LVPBC-0.14 composite. Table 2 gives the *R*_ct_ fitting values of the LVPBC-0.14 and pristine LVPC composites at different cycles, in view of the fact that *R*_ct_ is a major part of the internal resistance of a battery. As indicated in Table 2, *R*_ct_ of the LVPBC-0.14 composite is relatively stable, compared to the pristine LVPC composite at the different cycles. EIS results show that the LVPBC-0.14 composite can preserve good interfacial compatibility between the solid electrode and electrolyte solution, compared with the LVPC composite, which is a very important factor in improving the electrochemical properties of LVP.

**Figure 8:**
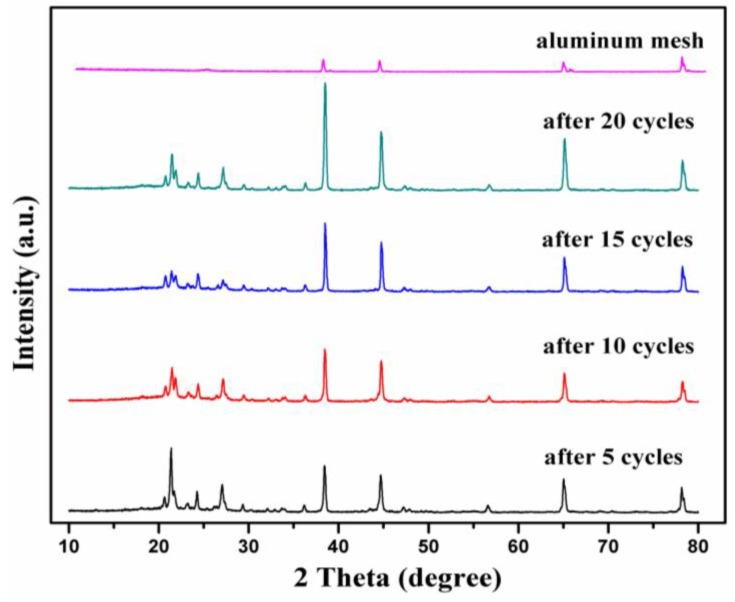
Ex-situ XRD patterns of LVPBC-0.14 electrode under fully discharged state at the different cycles.

To further confirm the excellent reversibility described above, ex-situ XRD characterization of the LVPBC-0.14 composites were carried out at a current density of 30 mA·g^−1^ after different cycles under the full discharged states, and the XRD patterns are shown in Figure 8. Compared with the uncharged LVPBC-0.14 composite, the main LVP diffraction peaks of the LVPBC-0.14 composite at the fully discharged states have emerged, in addition to the diffraction peaks of aluminum mesh. It is only the intensity of the diffraction peaks which change, in comparison with the reported ex-situ XRD result of the LVPC composite operated in the voltage range of 3.0–4.3 V [39]. Obviously, the structural reversibility of LVP in the voltage range of 3.0–4.8 V is slightly poorer than that of LVP in the voltage range of 3.0–4.3 V, because the former involves three Li^+^ ion intercalations/deintercalations. However, the four electrodes at the different cycles display the almost same diffraction patterns, disclosing that the LVPBC-0.14 composite with a stable crystal structure can be used as a high-performance energy storage cathode material for LIBs. 

## 4. Conclusions

In sum, a series of Br^−^-doped LVPC composites have been successfully synthesized by a rheological phase reaction method in this work. Galvanostatic charge/discharge measurements indicate that the LVPC-0.14 composite exhibits the best performance in both discharge capacity and cycling performance, in the voltage range of 3.0–4.8 V. The LVPC-0.14 composite reaches the initial discharge capacity of 178.33 mAh·g^−1^ with a capacity retention of 78.32% (higher than the pristine LVPC, ca. 72.32%), after 100 cycles at the current density of 30 mA·g^−1^, and still delivers a high initial capacity of 156.57 mAh·g^−1^, even at a high current density of 90 mA·g^−1^, with a small capacity decay rate of ca. 0.2% (lower than the pristine LVPC, ca. 0.263%) at each cycle for the first 100 cycles. The lower charge transfer resistance and structural stability are responsible for the outstanding electrochemical properties of the LVPC-0.14 composite. The strategy of anion doping in this study offers a convenient and effective technology for increasing the capacity and cycleability of LVP for LIBs, and more studies on the anion doping of LVP are in progress.

## Figures and Tables

**Table 1:** The lattice parameters of the Li_3_V_2_(PO_4_)_3−*x*_Br*_x_*/carbon (LVPBC) and pristine Li_3_V_2_(PO_4_)_3_/carbon (LVPC) composites.

Samples	*a* (Å)	*b* (Å)	*c* (Å)	β (°)	*V* (Å^3^)
LVPC	8.5120	8.6014	11.9180	89.3033	872.51
LVPBC-0.08	8.4698	8.5820	11.8675	89.2170	862.54
LVPBC-0.14	8.5314	8.5912	11.8956	89.4719	871.84
LVPBC-0.20	8.4836	8.5853	12.0350	89.5916	876.54
LVPBC-0.26	8.4545	8.5495	12.0230	88.6421	868.79

**Table 2:** *R*_ct_ fitting values of the LVPBC-0.14 and pristine LVPC composites at different cycles.

Samples	LVPBC-0.14 (10th)	LVPC (10th)	LVPBC-0.14 (20th)	LVPC (20th)
*R*_ct_ (Ω)	55.33	111	30.4	42.12
